# Prospective Evaluation of Radiocephalic Arteriovenous Fistula Outcomes in a High-Volume Centre: Patency, Complications, and Influencing Factors

**DOI:** 10.7759/cureus.105489

**Published:** 2026-03-19

**Authors:** Anum Arif, Anas Bin Saif, Raoon Khan, Assad Ali, Aima Sohail Asghar, Ahsin Manzoor Bhatti

**Affiliations:** 1 Department of Vascular Surgery, University Hospitals Birmingham NHS Foundation Trust, Birmingham, GBR; 2 Department of Vascular Surgery, Quetta Institute of Medical Sciences, Quetta, PAK; 3 Department of Family Medicine, University of Manitoba, Manitoba, CAN; 4 Department of Cardiac Surgery, Omar Hospital Lahore, Lahore, PAK; 5 Department of Family Medicine, University Medical Practice, Birmingham, GBR; 6 Department of Vascular Surgery, Combined Military Hospital Peshawar, Peshawar, PAK

**Keywords:** distal radio cephalic fistula, fistula surgery, hemodialysis access, permanant vascular access, primary patency, renal access, vascular access procedures

## Abstract

Introduction

Despite strong recommendations by the National Kidney Foundation (NKF) for the creation of radiocephalic arteriovenous fistula (RC AVF), most volume centres are reluctant due to contradictory complication rates and sustained patency. Factors influencing outcomes warrant attention, emphasising the importance of tailored approaches. Multicentre studies are recommended for broader validation of these findings. Therefore, the current study aimed to evaluate the outcome of RC AVF in our setup and to identify factors that influence its outcome.

Method

The prospective observational study was conducted at the Department of Vascular Surgery, Combined Military Hospital Lahore, over a year (October 2022-September 2023), and included 315 patients with RC AVF. Data were collected for demographic variables, comorbidities and fistula patency. The patients were further categorised into three subgroups on the basis of the location of RC AVF: snuff box, distal at the wrist, and proximal in the forearm. Follow-up evaluations were performed at one, six, and 12 weeks postoperatively. Statistical analysis was conducted using SPSS (IBM SPSS Statistics for Windows, IBM Corp., Version 22, Armonk, NY). The result shows that there was male predominance (75.2%).

Results

The mean age was 51.71 ± 14.94 years. Diabetes mellitus (40.6%) and hypertension (36.8%) were the most prevalent comorbidities. Distal radiocephalic arteriovenous fistula (DRC AVF) at the wrist was the most common access type (47.3%). Complications occurred in 9.2% of patients. Primary patency rates were 98.7% at one week, 92.1% at six weeks, and 90.5% at 12 weeks.

Conclusion

Radiocephalic fistula demonstrates favourable outcomes in our high-volume centre, with low complication rates and sustained patency. Factors influencing outcomes warrant attention, emphasising the importance of tailored approaches. Multicentre studies are recommended for broader validation of these findings.

## Introduction

The National Kidney Foundation (NKF) recommended fistula rates of ≥50% for incident (first placed access) and ≥40% for prevalent (previously surgically created access) patients undergoing haemodialysis [1]. Forearm autogenous arteriovenous (AV) access has been endorsed as the elemental choice for primary access in haemodialysis patients [2]. In contrast to AV grafts and central venous catheters, AV fistulas after maturation are associated with the lowest complication and infection rates, better long-term survival, and fewer interventions required to maintain long-term patency once successfully cannulated [2].

Radiocephalic AV fistula (RC AVF) is considered the gold standard venous access for patients requiring long-term haemodialysis, as it reduces the risk of steal syndrome compared with elbow fistulas and preserves proximal vessels for future use [3].

Despite these advantages, multiple studies have reported that distal RC AVFs at the wrist have significant primary failure rates due to early thrombosis or inadequate maturation for effective dialysis [4,5]. Poor maturation is reported to be associated with factors such as female gender, advanced age, and obesity [6]. Preoperative imaging predictors of maturation failure include radial artery diameter <2 mm, radial artery flow <40 mL/s, arterial calcification, and cephalic vein diameter <2.5 mm [7]. A major cause of impaired maturation in distal RC fistulas is juxta-anastomotic stenosis (JAS), the most common stenosis pattern in these fistulas, though also seen in brachiocephalic fistulas, where 22% demonstrate hemodynamically significant JAS [8,9].

Despite concerns regarding maturation failure, current guidelines still recommend constructing an RC AVF first, followed by a brachiocephalic fistula and then a brachiobasilic fistula if required [1]. This is largely because dialysis access-associated steal syndrome (DASS) is more common in proximal arm fistulas, occurring in 5-20% of cases, compared with approximately 1% in distal RC AVFs [10]. The risk increases to 25-81% in patients older than 60 years, females, those with peripheral vascular disease, or those with prior ipsilateral limb surgery [10]. Ali et al. reported that nearly one-third of RC AVFs fail within two years, with poorer outcomes in diabetic patients. Their study also found that forearm RC AVFs have more favourable outcomes than cubital fossa AVFs [5].

Although guidelines recommend distal access as the preferred site, surgical outcomes vary significantly between centres, leading some surgeons to avoid distal AVFs despite NKF Kidney Disease Outcomes Quality Initiative (KDOQI) recommendations due to inconsistent results at different centres [1,9].

Therefore, our study aimed to evaluate the outcome of RC AVF in our setup and to identify factors that influence its outcome.

## Materials and methods

This cross-sectional study was conducted in the Department of Vascular Surgery at Combined Military Hospital Lahore over a duration of one year, from October 2022 to September 2023, after taking approval from the Institutional Review Board (294/2021). A convenient random sampling technique was used. The sample size calculated with 95% confidence interval and 5% margin of error with the OpenEPI calculator (Dean AG, Sullivan KM, Soe MM, Emory University, Atlanta, GA) was 38.1. All the patients meeting the criteria of age ranging between 20 and 80 years, either gender, on haemodialysis due to end-stage renal disease, and with a radiocephalic fistula were recruited in the study after informed consent. Patients who refused to participate in the study, on table change in the surgical plan, were excluded.

The decision to make the site of the radiocephalic fistula either in the anatomical snuff box, distally in the wrist (distal radiocephalic arteriovenous fistula (DRC AVF)), or more proximally in the arm (proximal radiocephalic arteriovenous fistula (PRC AVF)) was made at the time of preoperative assessment with examination and duplex mapping in the access clinic and again on the day of surgery. 

Data were recorded prospectively by the vascular surgery team in terms of patients' demographics, anatomical location of radiocephalic fistula, duration of surgery and intraoperative complications. Patients were followed at one, six and 12 weeks postoperatively in the vascular surgery clinic. They were evaluated for whether the fistula was functional, postoperative complications, AVF thrombosis, infection, steal syndrome, neuropathy, aneurysm or pseudo-aneurysm, failure to mature and mortality of the patient. Patency was assessed by taking history for adequate dialysis, clinical examination and duplex scan in follow-up clinics by the Vascular Surgery team. Primary patency was stratified as a patent fistula without any further intervention, whereas secondary patency was stratified as the patency of the fistula after any intervention.

Data were collected in SPSS (IBM SPSS Statistics for Windows, IBM Corp., Version 22, Armonk, NY). Mean and SD were calculated for quantitative variables. Frequency and percentage were calculated for qualitative variables. p-value ≤ 0.05 was considered statistically significant.

## Results

A total of 315 patients were recruited. There was male predominance (n = 237, 75.2%). The mean age of the individuals was 51.71 ± 14.94 years, with an age range of 19 to 79 years. The most common comorbid disease was diabetes mellitus (n = 128, 40.6%), followed by hypertension in 116 (36.8%). Further demographic factors are shown in Table 1.

**Table 1:** Demographic characteristics of all patients (n = 315)

Variable	n (%)
Age group	
<20 years	2 (0.6)
21-30 years	34 (10.8)
31-40 years	41 (13.0)
41-50 years	80 (25.4)
51-60 years	77 (24.4)
61-70 years	55 (17.5)
71-80 years	26 (8.3)
Dialysis access	
Single-lumen catheter	160 (50.8)
Perma-catheter	83 (26.3)
None at time of surgery	54 (17.1)
Haemodialysis not started	18 (5.7)
Frequency of dialysis	
Not started yet	63 (20.0)
Once weekly	10 (3.2)
Twice weekly	189 (60.0)
Thrice weekly	53 (16.9)
Previous AVF	
Yes	55 (17.5)
No	260 (82.5)

The most commonly created access was DRC AVF in 149 (47.3%) patients, followed by PRC AVF in 120 (38.1%) and snuff box fistula in 46 (14.6%) patients. Primary AVF was created in 260 (82.5 %) of the patients.

**Figure 1:**
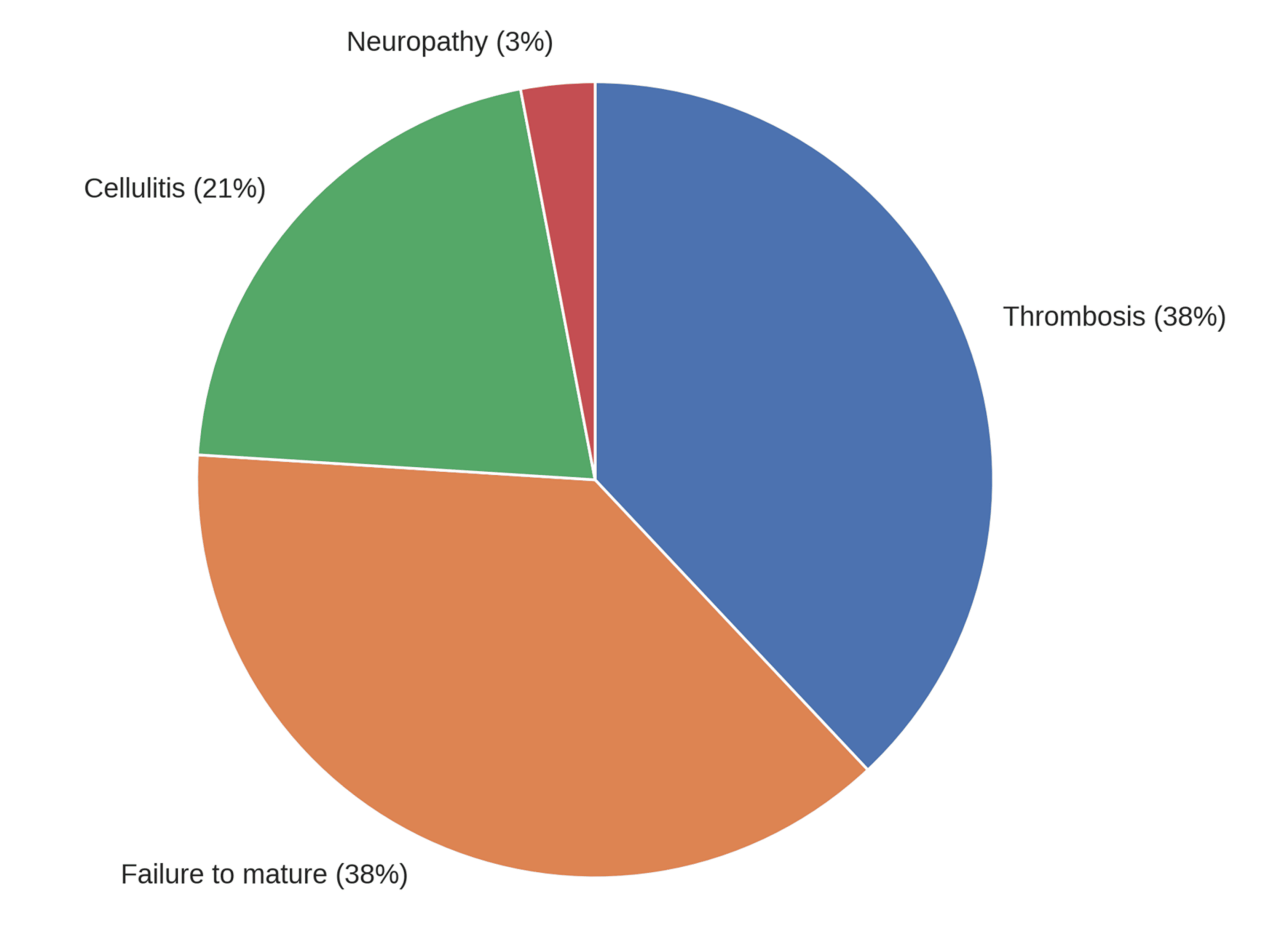
Postoperative complications (n = 29, 9.2%) This figure illustrates the distribution of postoperative complications among 29 patients. Thrombosis (38%) and failure to mature (38%) were the most common complications, followed by cellulitis (21%) and neuropathy (3%). Image made using Microsoft Word (Microsoft® Corp., Redmond, WA).

We found 29 (9.2%) patients had postoperative complications, with failure to maturation (11, 38%) and later thrombosis (11, 38%) being the leading causes, as shown in Figure 1. No shared factors were found for the complications.

**Figure 2:**
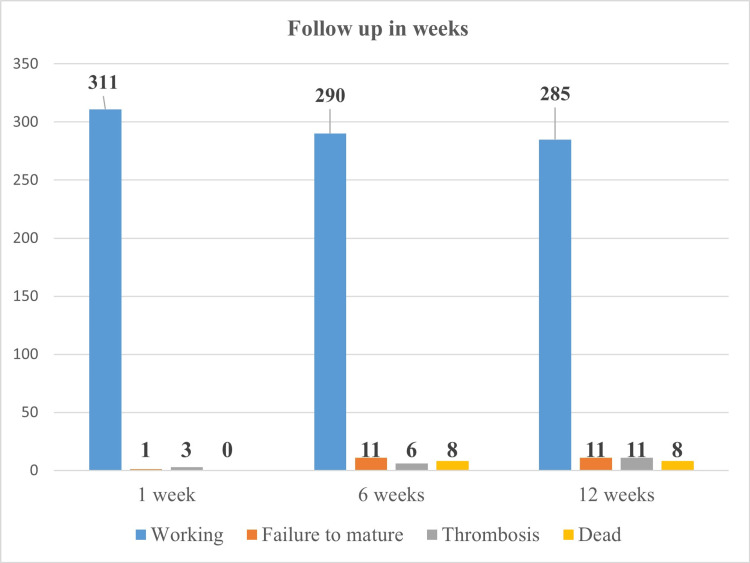
Outcome of follow-up at one week, six weeks, and 12 weeks (n = 315) This figure shows outcomes at one, six, and 12 weeks. Most fistulas were working at all time points, with gradual increases in thrombosis and stable failure-to-mature rates. Mortality reached eight patients by six weeks and remained unchanged at 12 weeks. Image made using Microsoft Word (Microsoft® Corp., Redmond, WA).

Follow-up at one, six, and 12 weeks is shown in Figure 2.

Primary patency (assessed by taking history for adequate dialysis, clinical examination, and duplex scan in follow-up clinics by the Vascular Surgery team) at one week was 98.7%, at six weeks it was 92.1%, and at 12 weeks follow-up it was 90.5%. None of the patients in the cohort had any secondary intervention, and therefore, secondary patency could not be calculated. We found that 285 out of 315 (90.47) fistulas were functional at 12-week follow-up.

Further association between outcome at 12 weeks and various demographic factors is shown in Table 2.

**Table 2:** Association between 12-week AVF outcome and demographic and clinical variables (n = 315)

Variable	Category	Working n (%)	Failure n (%)	Thrombosis n (%)	Death n (%)	p-value
Age group (years)	<20 (n = 2)	2 (100%)	0	0	0	0.041
	21-30 (n = 34)	32 (94.1%)	0	2 (5.9%)	0	-
	31-40 (n = 41)	35 (85.4%)	2 (4.9%)	4 (9.8%)	0	-
	41-50 (n = 80)	66 (82.5%)	6 (7.5%)	2 (2.5%)	0	-
	51-60 (n = 77)	69 (89.6%)	3 (3.9%)	3 (3.9%)	6 (7.8%)	-
	61-70 (n = 57)	55 (96.5%)	0	0	2 (3.5%)	-
	>70 (n = 26)	26 (100%)	0	0	0	-
Gender	Male (n = 237)	214 (90.3%)	9 (3.8%)	9 (3.8%)	5 (2.1%)	0.75
	Female (n = 78)	71 (91.0%)	2 (2.6%)	2 (2.6%)	3 (3.8%)	-
Comorbidities	DM (n = 116)	106 (91.4%)	5 (4.3%)	5 (4.3%)	0	-
	HTN (n = 128)	122 (95.3%)	2 (1.6%)	4 (3.1%)	0	-
	DM + HTN (n = 65)	51 (78.5%)	4 (6.2%)	2 (3.1%)	8 (12.3%)	-
	IHD (n = 2)	2 (100%)	0	0	0	-
	DM + HTN + IHD (n = 4)	4 (100%)	0	0	0	-
Dialysis frequency	Not on dialysis (n = 63)	55 (87.3%)	4 (6.3%)	2 (3.2%)	2 (3.2%)	-
	Once weekly (n = 10)	8 (80.0%)	1 (10.0%)	1 (10.0%)	0	-
	Twice weekly (n = 189)	175 (92.6%)	5 (2.6%)	5 (2.6%)	4 (2.1%)	-
	Thrice weekly (n = 53)	47 (88.7%)	1 (1.9%)	3 (5.7%)	2 (3.8%)	-
Previous AVF	Yes (n = 55)	55 (100%)	0	0	0	-
	No (n = 260)	230 (88.5%)	11 (4.2%)	11 (4.2%)	8 (3.1%)	-
Type of fistula	Snuff box (n = 46)	40 (87.0%)	2 (4.3%)	2 (4.3%)	2 (4.3%)	0.647
	Distal RCF (n = 149)	133 (89.3%)	6 (4.0%)	6 (4.0%)	4 (2.7%)	<0.001
	Proximal RCF (n = 120)	112 (93.3%)	3 (2.5%)	3 (2.5%)	2 (1.7%)	0.893

The DRC AVF had the highest primary patency at 12 weeks (n = 133, 42.2%). Chi-square test was used, and the p-value was 0.89.

## Discussion

The prevalence of chronic kidney disease in Pakistan ranges from 12.5% to 25.5% [11,12]. A considerable number of these patients will require renal replacement therapy in the coming years. Kidney transplantation remains limited due to the scarcity of transplant centres, most of which operate only live donor programs; therefore, haemodialysis remains the predominant modality of renal replacement therapy [11]. Although central venous catheters are used in emergency situations, AV fistulas (AVFs) remain the preferred access. International guidelines recommend creating an AVF once the GFR falls below 20 mL/min; however, in Pakistan, this practice is uncommon, largely due to patient apprehension and lack of awareness [12].

This trend was evident in our study, where 20% of patients were not yet on dialysis at the time their access was created. Nasir Mahmood et al. similarly reported that only 20% of patients had a mature AVF at dialysis initiation, with most referrals coming from nephrologists rather than general physicians [13]. Globally, the percentage of patients who receive AV access before starting haemodialysis varies widely, from less than 10% in some countries to over 80% in others. In the United States, the Fistula First Breakthrough Initiative significantly increased AVF use, and more than 80% of patients initiated haemodialysis with an AV access [14].

There was a male predominance in our study (75.2%), consistent with both local and international literature. Khan et al. reported 71% male patients [15], while international data also show a male majority [2]. The mean age in our study was 51 years. International studies typically report older populations, with mean ages around 61 years [2]. Pakistani cohorts, however, consistently show younger patients [4,5,13], likely due to unhealthy lifestyles, poor medication adherence, and a high burden of diabetes [16].

Diabetes mellitus was the most common comorbidity in our cohort (40.6%), followed by hypertension (36.8%). While hypertension is a major global cause of ESRD, Pakistani data consistently show diabetes as the leading contributor to renal failure [11,12]. Whether diabetes is the primary cause or develops secondary to renal dysfunction remains debated.

KDOQI guidelines emphasise creating the most distal fistula possible when suitable [1]. We offered snuff box AVFs-the most distal option-to 46 patients (14.6%). This technique requires strong clinical judgment in addition to radiological assessment. Literature on snuff box AVFs in Pakistan is scarce, but in the United States, they account for approximately 20% of haemodialysis access [12].

Many ESRD patients lack adequate distal veins and have diseased radial arteries. In our study, the three-month primary patency rate was 90.5%, higher than other local studies reported 50-80% [5,13,15]. Schinstock et al. reported three-month primary and secondary patency rates of 67% and 92%, respectively [14]. Our superior outcomes may reflect high surgical volume, standardised preoperative assessment, use of microsurgical instruments, and adherence to meticulous postoperative surveillance.

Failure to mature and thrombosis were the most common complications in our study, each occurring in 3.5% of patients. This aligns with published literature. Al-Shameri et al. reported thrombosis as the most common complication (13%) [17], while Azeem et al. found failure to mature to be the most frequent issue (12.4%) [18].

Subgroup analysis revealed the poorest patency in patients aged 30-60 years. Additionally, patients with both hypertension and diabetes had the highest failure rate (21.6%) at 12 weeks. These findings differ from previous studies. A Turkish study found no significant difference in patency between diabetic and non-diabetic patients [19], whereas Schinstock et al. reported increased failure risk among diabetics [14]. The poorer outcomes in middle-aged patients with dual comorbidities may reflect more aggressive disease progression.

This study has limitations. It was conducted at a single centre, limiting generalizability. The follow-up period was short, and financial considerations were not assessed. A multicentre study with longer follow-up is recommended to validate these findings.

## Conclusions

Despite concerns regarding primary failure due to inadequate maturation or thrombosis, DRCF remains the gold standard, as emphasised by KDOQI guidelines. The study reported a 90.5% primary patency rate at three months, surpassing outcomes from other local studies, underscoring the benefits of standardised protocols, preoperative imaging, and meticulous postoperative care in a high-volume setup. Diabetes and hypertension were prominent comorbidities, with failure to mature and thrombosis being the most frequent complications. These findings validate the utility of DRCF in minimising complications like steal syndrome and preserving proximal vessels for future use.

## References

[1] Lok CE, Huber TS, Lee T (2020). KDOQI clinical practice guideline for vascular access: 2019 update. Am J Kidney Dis.

[2] Bylsma LC, Gage SM, Reichert H, Dahl SL, Lawson JH (2017). Arteriovenous fistulae for haemodialysis: a systematic review and meta-analysis of efficacy and safety outcomes. Eur J Vasc Endovasc Surg.

[3] Elfström J, Lindell Å (1994). Limitations of arteriovenous fistulae in the cubital fossa. Scand J Urol Nephrol.

[4] Gosadi IM, Daghriri KA, Majrashi AA (2020). Lifestyle choices and prevalence of chronic noncommunicable diseases among primary healthcare physicians in the Jazan Region, Saudi Arabia. J Family Med Prim Care.

[5] Nawaz S, Ali S, Shahzad I, Baloch MU (2013). Arterio venous fistula experience at a tertiary care hospital in Pakistan. Pak J Med Sci.

[6] Miller CD, Robbin ML, Allon M (2003). Gender differences in outcomes of arteriovenous fistulas in hemodialysis patients. Kidney Int.

[7] Beathard GA (2005). An algorithm for the physical examination of early fistula failure. Semin Dial.

[8] Badero OJ, Salifu MO, Wasse H, Work J (2008). Frequency of swing-segment stenosis in referred dialysis patients with angiographically documented lesions. Am J Kidney Dis.

[9] Quencer KB, Arici M (2015). Arteriovenous fistulas and their characteristic sites of stenosis. AJR Am J Roentgenol.

[10] Mishra B (2021). Comparison of distal radiocephalic fistula vs proximal radiocephalic fistula. J Family Med Prim Care.

[11] Alam A (2022). Health policy for kidney diseases in Pakistan- a need of time to consider. Pak J Kidney Dis.

[12] Ahmed J, Azhar S, Ul Haq N (2022). Awareness of chronic kidney disease, medication, and laboratory investigation among nephrology and urology patients of Quetta, Pakistan. Int J Environ Res Public Health.

[13] Nasir Mahmood S, Naveed Mukhtar K, Iqbal N, Umair SF (2013). Pre dialysis care and types of vascular access employed in incident hemodialysis patients: a study from Pakistan. Pak J Med Sci.

[14] Schinstock CA, Albright RC, Williams AW (2011). Outcomes of arteriovenous fistula creation after the Fistula First Initiative. Clin J Am Soc Nephrol.

[15] Khan F, Soomro N, Ahmed I, Rehman SU, Qamar U, Ahmed Z (2022). Clinical characteristics and outcome of arteriovenous fistula among patients undergoing haemodialysis with end stage renal disease. Pak J Med Health Sci.

[16] Saqlain M, Riaz A, Malik MN, Khan S, Ahmed A, Kamran S, Ali H (2019). Medication adherence and its association with health literacy and performance in activities of daily livings among elderly hypertensive patients in Islamabad, Pakistan. Medicina (Kaunas).

[17] Al-Shameri I, KhudaBux GM, Al-Ganadi A (2021). Prospective evaluation of factors associated with arteriovenous fistula primary failure and complications in hemodialysis patients: a single center-study. Cardiol Vasc Res.

[18] Azeem SM, Ehsan O, Khan MI (2022). Patency and complications of arterio-venous fistula created in pre- and post-dialysis settings. J Coll Physicians Surg Pak.

[19] Kocaaslan C, Kehlibar T, Coşkun G (2015). Comparison primary failure and primary patency rates of distal radiocephalic arteriovenous fistulas in diabetic and non diabetic patients. Turk Klin Kardiyovask Sci.

